# 1635. Assessing the Potential Reduction in Morbidity and Mortality Attainable by Delayed Influenza Vaccine Strain Selection

**DOI:** 10.1093/ofid/ofad500.1469

**Published:** 2023-11-27

**Authors:** Stephen M Kissler, Keya Joshi, Nicolas Van de Velde, Nevena Vicic, Yoonyoung Park, Deborah Rudin, Parinaz Ghaswalla

**Affiliations:** University of Colorado Boulder, Boston, Massachusetts; Moderna, Inc, Cambridge, Massachusetts; Moderna, Inc., Cambridge, Massachusetts; Moderna, Inc, Cambridge, Massachusetts; Moderna, Inc, Cambridge, Massachusetts; Moderna, Inc, Cambridge, Massachusetts; Moderna, Inc., Cambridge, Massachusetts

## Abstract

**Background:**

To allow time for production, seasonal influenza vaccine target strains must be chosen up to 8 months before the start of the influenza season. Antigenic drift during these months can lead to mismatch between the circulating and vaccine strains, negatively impacting vaccine effectiveness (VE). mRNA vaccines can be produced more quickly than standard vaccines, raising the possibility of delaying influenza vaccine strain selection by up to 3 months, thus potentially improving VE. This is especially important for the H3N2 subtype, which is generally more severe than other subtypes and causes most human infections. The potential reductions in morbidity and mortality attainable by delayed influenza vaccine strain selection, especially for H3N2 subtype, have not yet been examined.

**Figure:**
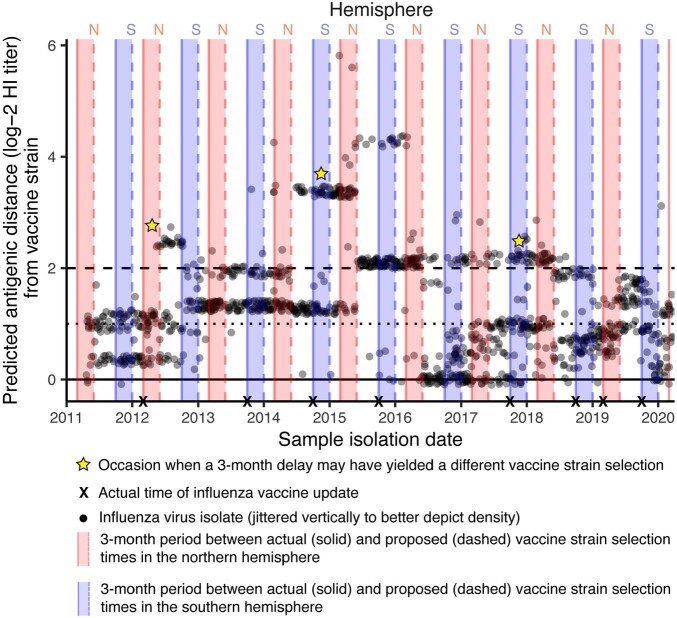

**Methods:**

We assessed the match between circulating and vaccine strains (only H3N2 subtype) using influenza phylogenetic data and associated predictions of antigenic distance (difference in log-2 HI titer) from 2011-2019 obtained from GISAID and analyzed using *NextStrain*. We then developed a mathematical model to estimate the potential reduction in symptomatic cases, hospitalizations, and mortality attainable by a 3-month delay in influenza vaccine strain selection. A direct estimation of final epidemic size, trained to historical data on influenza burden from the US Centers for Disease Control and Prevention, was used to parametrize the model.

**Results:**

In 2011-2019, a 3-month delay in strain selection could have detected the emergence of antigenically distinct H3N2 strains ( > 2 predicted log-2 HI titer difference from the vaccine) on 3 occasions: Mar (June) 2012, Sep (Dec) 2014, and Sep (Dec) 2017 (**Figure**). Improved strain selection in 2012 and 2017 may have had only a limited impact: VE in the NH 2012-13 season remained high despite the suboptimal match, and circulation of influenza in the SH in 2018 was low. However, in 2014, improved strain selection could have yielded substantial benefits, with model-estimated reductions in hospitalizations and mortality between 15-20%.

**Conclusion:**

Delayed seasonal influenza vaccine strain selection, made possible by mRNA vaccine technology, could improve vaccine strain match and potentially yield reductions in hospitalizations and mortality in some seasons.

**Disclosures:**

**Stephen M. Kissler, PhD**, ModernaTx: Advisor/Consultant **Keya Joshi, PhD**, Moderna, Inc.: Employee|Moderna, Inc.: Stocks/Bonds **Nicolas Van de Velde, PhD**, Moderna, Inc.: Salary|Moderna, Inc.: Stocks/Bonds **Nevena Vicic, MSc**, Moderna, Inc.: Employee|Moderna, Inc.: Stocks/Bonds **Yoonyoung Park, ScD**, Moderna, Inc.: Employee|Moderna, Inc.: Stocks/Bonds **Deborah Rudin, MD**, Moderna, Inc.: Employee|Moderna, Inc.: Stocks/Bonds **Parinaz Ghaswalla, PhD**, Moderna, Inc: Employee|Moderna, Inc: Stocks/Bonds

